# Radiographic assessment of the quality of post and core restorations performed by general dental practitioners in Saudi Arabia

**DOI:** 10.1016/j.heliyon.2024.e31637

**Published:** 2024-05-22

**Authors:** Moazzy I. Almansour, Ahmed A. Madfa, Alanoud N. Alotaibi, Rawan T. Alturki, Ahad F. Alshammari

**Affiliations:** a Department of Restorative Dental Science, College of Dentistry, University of Ha'il, Ha'il, Kingdom of Saudi Arabia; b Dentist, Private Sector, Ha'il, Kingdom of Saudi Arabia

**Keywords:** Post and core restorations, Dental practitioners, Quality, Endodontically treated teeth

## Abstract

**Background:**

Dentists utilize various techniques and materials for post and core restoration of endodontically treated teeth, which remains a noteworthy health concern and can be addressed through interdisciplinary strategies to enhance outcomes. Therefore, this study aimed to evaluate the technical quality of the residual gutta-percha (GP) and posts by analysing the periapical radiographs of endodontically restored teeth.

**Methods:**

A total of 594 periapical digital radiographs were evaluated for tooth type, post material, post shape, design, diameter, length, residual GP, GP length, GP tapering, GP homogeneity, and final restoration. Frequency distribution and cross-tabulation of the variables were performed.

**Results:**

Maxillary molars had the highest frequency of restorations, including posts, accounting for 27.4 %, followed by maxillary premolars (25.4 %) and maxillary anterior teeth (19.2 %). The prefabricated metallic posts were most commonly used (81.0 %), among these, 50.4 % were screwed posts. Tapered posts were the most commonly used (65.6 %). The percentage of posts with an acceptable length was 58.2 %. The most commonly used posts exhibited a width of one-third of the root diameter, accounting for 87.0 %. Suitable GP lengths were observed in 61.1 % of the restorations, and 62.8 % demonstrated adequate GP homogeneity. The findings of this study revealed that crown restoration accounted for 42.6 % of the studied sample.

**Conclusions:**

The technical quality of the remaining GP after restoration was comparable to that of previous epidemiological investigations. However, the technical quality of the post was deemed suboptimal.

## 1 Background

Restoring endodontically-treated teeth is an essential aspect of dental care and involves diverse treatment options with varying complexity [1]. Protecting the surrounding tooth structure and minimising the risk of further damage is imperative following endodontic therapy. Endodontically treated teeth with extensive loss of supporting structures such as the marginal ridge often require restorations that cover the cusps, which minimises occlusal stress and reduces the risk of coronary fractures [2,3].

Advancements in the restoration of endodontically treated teeth have made substantial progress in recent decades; however, there remains a contentious debate on this topic [[4], [5], [6]]. However, the process of restoring teeth that have undergone endodontic therapy is challenging because of the considerable loss of tooth structure [7]. Furthermore, restorative materials must possess adequate efficacy for inhibiting bacterial microleakage and minimising the probability of root fractures [8]. Several factors should be considered when deciding on post-endodontic therapy, such as the extent of the remaining tooth structure, position of the tooth in the dental arch, and functional and aesthetic concerns [9]. Evaluating these parameters is crucial because the lack of adequate restorative therapy is a potential factor implicated in treatment failures [10]. The substantial scale of this loss requires the adoption of a core build-up manufacturing procedure that largely focuses on safeguarding prosthetic restoration. Furthermore, the need for an intraradicular post to effectively stabilise core filling may be determined by the magnitude of the remaining coronary structures [11].

Clinical examinations and diagnostic periapical radiography play crucial roles in determining the type, length, and diameter of the post [12]. Retention is lower in tapered posts than in parallel posts, while maintaining clinically appropriate levels of retention for both post types. Compared to tapered posts, parallel-sided posts exhibit a more uniform stress distribution and offer enhanced resistance to both tensile and shear stresses [13]. The length of the post should be equal to or greater than the height of the clinical crown or half the length of the root [14]. The ideal length of a post should be approximately two-thirds of the root length, while also leaving a 3–5 mm space of GP apically [15]. Insufficient post length may result in less retention and increased stress, potentially leading to root fractures. The relative stresses in the cervical region were more influenced by post length than by post diameter. Specifically, short, broad posts resulted in higher stress concentrations in the cervical region. Nevertheless, the placement of posts beyond two-thirds of the root results in elevated stress levels in the apical region [16]. Trabert et al. proposed that the diameter of the post should not exceed one-third of the root diameter at any given site [17]. Root size relative to post size and root fractures exhibit a direct correlation, indicating that extensive tooth preparations may increase the probability of root fractures [18]. Ideally, there should be no space between the post and remaining GP. This space has the potential to harbour bacteria, which can negatively impact the outcome of endodontic treatment and increase the likelihood of developing periapical lesions [19]. The recommended remaining GP length necessary to preserve apical integrity and ensure a suitable apical seal should be within 3–5 mm [20]. Retaining GP >5 mm while maintaining the optimal length of the post presents no potential disadvantages [21].

Extensive studies have been conducted on the biochemical underpinnings of endodontic therapy. The technical quality of restorative techniques and their influence on patient prognosis have gained considerable attention. Ineffective or unsuccessful techniques may result in the migration of microorganisms and their byproducts to the periarticular region and adjacent alveolar bone [22]. The present study aimed to evaluate the technical quality of the remaining GP and post and core restorations done by general dentists in the Hail region in patients with endodontically treated teeth using periapical radiographs.

## 2 Methods

To analyse the post and core restorations performed by the general dentists, a retrospective cross-sectional observational investigation was conducted in the Hail province. The study was formally approved by the Medical Ethics Committee of the University of Hail (No.: H-2022-328) and conducted in accordance with the tenets of the Declaration of Helsinki. Owing to the retrospective nature of the study design, the requirement for informed consent was waived by the College of Dentistry's ethical council.

The sample size was determined using Cochran's formula: N = (Zα2 × P (1-P))/D2, where Zα represents the critical value of the normal distribution at α/2 (1.96), D represents the desired degree of precision, and P represents the technical quality of the root canal fillings (69 %) as determined by a previous study conducted in the Eastern Province of Saudi Arabia [23]. The recommended sample size was 323. The final sample comprised 594 digital periapical radiographs.

The dataset comprised 594 digital periapical radiographs of the posts used to restore endodontically treated teeth. These radiographs were collected from general dentists between September 2012 and April 2023. Data collection relied on information derived from patient records and a radiographic program known as the CSR4 software, developed by Carestream Dental LLC in the United States. The data assigned to each sample was documented using a form specifically created for this investigation. Samples were excluded if a radiograph of the post was not accessible or if the radiograph obtained was of insufficient diagnostic quality. The FOCUS™ intraoral X-ray imaging device, manufactured by KaVo™ in Finland, was utilised in this study. The dental unit was capable of generating dental images of superior quality using a digital sensor. The sensor employed in this study was of the RVG type and utilised a film XCP holder for precise positioning adjustments. The exposure settings for the adult mode typically involved a fixed kilovoltage (kV) of either 60 or 70 kV along with an adjustable exposure period ranging from 0.02 to 3.2 s. The duration of exposure was determined by factors such as the specific tooth being imaged, size of the patient, and selected mode of exposure. Radiographs of the patients were observed on the R4 system using the "Kodak Dental Imaging" software. The data were collected and recorded in an Excel spreadsheet by two dentists. The description "ideal" was assigned to data that adhered to the criteria outlined in Table 1, Table 2.

**Table 1:** Intra-radicular post parameters.

Parameters	Type
Post material	Metallic, Non-Metallic, Amalgam post
Post shape	Taper, Parallel
Post design	Screwed, Cemented

**Table 2:** Parameters recorded on endodontically treated teeth.

Definition	Criteria	Parameters
Quality of root canal treatment		
Length	Acceptable	Root canal filling 0–2 mm short from the radiographic apex.
	Unacceptable	Root canal filling beyond the radiographic apex or root canal filling >2 mm from the radiographic apex.
Homogeneity	Acceptable	Homogeneous root canal filling, good condensation, no visible voids.
	Unacceptable	Non-homogeneous root canal filling, poor condensation or voids present.
Taper	Acceptable	Consistent and uniform taper from the coronal to apical area with a reflection of the original shape of the canal.
	Unacceptable	Non-consistent taper.
Post diameter	Acceptable	Not exceed 1/3 of the root diameter at any given point along the root length.
	Unacceptable	If the post is more than one third of the root diameter or less than one third of the root diameter.
Post length	Acceptable	Equal to the length of the clinical crown or 2/3 length of the canal
	Unacceptable	If the post length is less than 2/3 of the canal length or more than 2/3 of the canal length.
GP remanent	Acceptable	3–5 mm
	Unacceptable	If the GP remanent is more than 5 mm or the GP extruded through the root apex
Coronal status		
Intra-coronal restoration	Acceptable	Any permanent restoration that appeared intact radiographically.
	Unacceptable	Any permanent restoration with detectable radiographic signs of overhangs, open margins, or recurrent caries.
extra-coronal restoration	Crown	

The subsequent data were used as evaluation parameters.

• Tooth type.
• Post material
• Post shape
• Post design
• Post diameter
• Post length
• Residual GP
• GP length
• GP tapering
• GP homogeneity
• Type of final restoration (crown or restoration)

Calibration training was undertaken prior to the evaluation. The examiners, ANA, RTA, and AFA underwent calibration procedures under the supervision of MIA and AAM in accordance with the established standards and variations outlined before commencement of the experimental reading. A random selection was made for 20 % of the sample, which was reviewed by the examiners. The agreement between observers was evaluated by calculating the kappa coefficient, resulting in a value of 0.95. The observers conducted a concurrent assessment, and any discrepancies were handled by conversation and mutual agreement. After the initial review, the same examiner conducted a further analysis two weeks later. The examiners were blinded during the second analysis. Approximately 20 % of the sample was used for this purpose, with the aim of evaluating intra-observer reliability. The level of agreement among observers, as measured by intra-observer agreement, was 0.90.

All statistical analyses were performed using the Statistical Package for the Social Sciences (SPSS) software (IBM Corp., Chicago, IL, US). Frequency distribution and cross-tabulation of the variables were performed. Post parameters, root canal treatment quality, and coronal status were evaluated. The level of significance was set at 5 % (α = 0.05).

## 3 Results

**Fig. 1:**
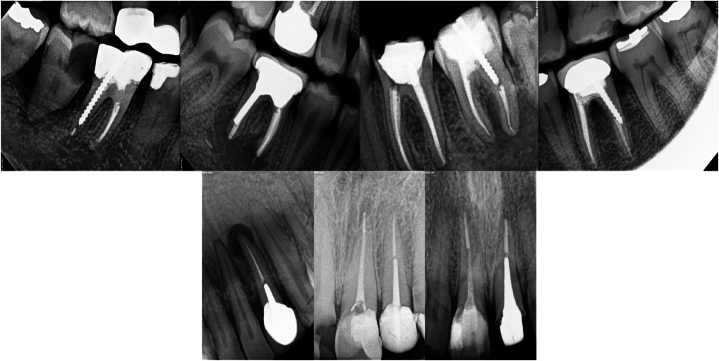
Some endodontically treated teeth restored with various post and core restoration reported in this study.

Table 3 summarizes the frequencies and percentages of several factors, including the arch, tooth type, post type, post width, post length, coronal restorations, and GP condition. The frequency of post and core restorations was the highest for maxillary molars (27.4 %), followed by the maxillary premolars (25.4 %), and maxillary anterior teeth (19.2 %). In contrast, in the mandibular anterior teeth, the percentage of post and core restorations was lower, accounting for only 3.5 % of the analyzed samples. The findings of this study indicated that the most commonly used posts were prefabricated metallic posts, accounting for 81.0 % of the samples; among these, screwed and cemented posts constituted 50.4 % and 49.6 % of the samples, respectively. The tapered post was the most frequently used, accounting for 65.6 %, whereas the parallel post was used in 34.4 % of the analyzed samples. Fig. 1 displays different post and core restorations utilised for the restoration of teeth that have undergone endodontic treatment.

**Table 3:** Quality of root canal fillings based on length, density, and taper criteria.

Variables		Frequency	Percent
**Tooth type**	Maxillary anterior	114	19.2
	Mandibular anterior	21	3.5
	Maxillary premolar	151	25.4
	Mandibular premolar	113	19.0
	Mandibular molar	32	5.4
	Maxillary molar	163	27.4
**Post material**	Metallic	481	81.0
	Non-Metallic	59	9.9
	Amalgam post	54	9.1
**Post shape**	Taper	354	65.6
	Parallel	186	34.4
**Post design**	Screwed	272	50.4
	Cemented	268	49.6
**Post diameter**	Acceptable	517	87.0
	Unacceptable	77	13.0
**Post length**	Acceptable	346	58.2
	Unacceptable	248	41.8
**GP remanent**	Acceptable	554	93.3
	Unacceptable	40	6.7
**GP length**	Acceptable	363	61.1
	Unacceptable	231	38.9
**GP tapering**	Acceptable	421	70.9
	Unacceptable	173	29.1
**GP homogeneity**	Acceptable	373	62.8
	Unacceptable	221	37.2
**Coronal seal**	Crown	253	42.6
	Restoration	341	57.4

Radiographs of the periapical area were analyzed using the study criteria to determine the optimal post length. In approximately 58.2 % of samples, the posts were of the same length or longer than the crown, meeting the adequacy criterion. Similarly, assessment of the remaining length of the root filling indicated satisfactory root filling in 93.3 % of the samples. The most commonly utilised posts exhibited a width of one-third of the root diameter, accounting for 87.0 %, followed by two-thirds width of the reference post, which constituted 13.0 %. Approximately 61.1 % of the samples exhibited a suitable GP length, whereas 62.8 % demonstrated adequate GP homogeneity. Our study findings revealed that crown restorations accounted for 42.6 %, and coronal restorations constituted the remaining 57.4 %.

## 4 Discussion

The efficacy of post and core restorations has been evaluated using several criteria. The majority of criteria primarily rely on radiographic assessment, either on its own or in conjunction with clinical examination [24,25]. This study aimed to ascertain the technical quality of the remaining GP and posts used to restore endodontically treated teeth. The study findings were mainly derived from the evaluation of radiographic data using periapical digital radiographs. The prevalence of post restorations was more frequent in the jaw arch than in the mandibular jaw, which is consistent with those of Jamani et al. [26] and Al-Hamad et al. [27], who reported that post and core restorations were more common in the maxillary arch.

Our findings that 87.0 % of the study sample had an acceptable diameter were comparable with the suggested diameter of clinically appropriate posts, which is one-third of the root diameter, as previously published [28,29]. The observed outcome can be attributed to the limited proficiency of recently general practitioners in preparing root canals for post placement. Although retention was lower in tapered posts than in parallel posts, both types demonstrated clinically appropriate levels of retention [30]. Approximately 65.6 % of the posts in the included samples were tapered. This aligns with the results of Al-Hamad et al. [27] and is more similar to the findings of Jamani et al. [26], who reported the use of a tapered post in 74 % of patients. The widespread commercial availability of tapered post systems is responsible for these outcomes. This phenomenon may be attributed to their inclination towards a more conservative approach in relation to radicular dentin. In the current investigation, metallic posts were utilised in most patients (81.0 %), whereas prefabricated non-metallic posts were employed in only 9.9 % of patients. Furthermore, Nimigean et al. [28] reported similar findings, indicating that metallic posts were used in 69.6 % of patients included in their analysis.

Screwed dental posts are not preferred due to their disadvantages as the higher incidence of root fractures lowers the survival rate significantly. Owing to root fracture, which is notably higher than the 3 % failure rate observed with cemented posts, these posts are often regarded as potential contributors to stress and are associated with a failure rate of 7 % [[31], [32], [33]]. Schmitter et al. [34] demonstrated a markedly superior survival rate of fibre posts in comparison to metal screw posts. Previous studies have shown that the use of screw posts can lead to fractures, which should be avoided because they cause stress concentration and increase the risk of root fracture [34,35]. However, in this study, the using screwed posts among dental practitioners were used in 50.4 % of the samples. The survival rates of teeth restored using tapered threaded posts are lower compared to teeth treated with cemented posts [36,37]. Because non-metallic materials exhibit physical properties comparable to those of natural dentin, such as modulus of elasticity, extensive utilisation of non-metallic materials is advocated whenever feasible [28,29].

In order to achieve the best possible balance between post length and important variables, including the apical seal, strength, or integrity of the remaining roots, the optimum post length should be as long as feasible [27,38,39]. According to McComb [30], it is advised that the length of the post should be half the length of the root, measured from the midpoint between the apex and alveolar crest. Additionally, there should be a leave 4–5 mm of GP at the apex. The current investigation found that 58.2 % of the samples had an acceptable post length, which is consistent with the findings published by Al-Hamad et al. [27]. In relation to the RCT, the recommended length of the remaining GP that should be inserted at the left apical end of the posts to ensure the integrity and appropriate sealing of the apex should be between 3 and 4 mm or 3–5 mm [26,27].

Preservation of the apical seal is a crucial factor in post space design, along with the quantity and quality of the remaining root filling and root canal therapy. A substantial proportion (93.3 %) of the analyzed samples in our study exhibited a 3–5 mm residual GP. This finding contrasts with the results reported by Meshni et al. [40], in which only 55.7 % of the examined cases showed a similar amount of residual GP. Furthermore, Mathar and Almutairi [41] reported an even lower percentage (28 %) of cases with 3–5 mm of remaining GP. Roots with posts and a remaining root filling length of less than 3 mm displayed a statistically significant increase in the occurrence of apical periodontitis [42]. However, the present study did not yield similar findings regarding remaining root filling quality. The observation that only 6.7 % of the samples examined in this study had root fillings with a surviving length of less than 3 mm supports this explanation. Furthermore, the limited representation of such cases in the study population had a negligible effect on the overall findings. To substantiate clinical decision making with scientific evidence, additional research that spans a prolonged period is recommended to enable data observation. A comprehensive evaluation of clinical and radiographic factors is crucial for achieving favourable outcomes in the restoration of endodontically treated teeth using post and cores.

Regarding the condensation and density of the remaining GPs, 37.2 % had an inadequate density. This observation contradicted the results reported by Naumann et al. [43]. The aforementioned findings clearly illustrate that the dentist responsible for performing the root canal procedure also performed the post space preparation, demonstrating a high level of expertise in identifying the presence of root curvatures and performing additional apical preparations.

Inadequate treatment increases the likelihood of persistent or new intraradicular infection, which is the major contributing factor for post-treatment apical periodontitis [44]. The assessment of treatment quality was based on the quality of the dental fillings, as assessed by radiographic analysis. Compared to inadequately treated teeth, teeth classified as effectively treated had a notably superior condition. The probable cause of this disparity is that a substantial number of teeth classified as adequately treated were not satisfactorily treated. This is attributed to the widely acknowledged constraints of radiography in assessing the efficacy of endodontic therapy [45]. Furthermore, the evaluation of teeth was based solely on the quality of the filling without any accompanying details regarding the disinfection protocols employed.

Coronal restorations have been recognized as a possible factor that could impact periarticular diseases [46,47]. The study findings indicated that teeth with sufficient coronal restorations exhibited significantly improved periarticular conditions compared to teeth with insufficient or absent restorations. The most favourable results were obtained in teeth that received sufficient endodontic therapy and appropriate coronal restoration. These teeth exhibited a considerably higher health rate than the other combinations, except for teeth that received sufficient treatment but had insufficient or absent repair. Therefore, achieving the most favourable result is contingent on the tooth being appropriately managed as a unified entity, encompassing both endodontic therapy and coronal restoration, in accordance with established norms. The primary purpose of coronal restorations in endodontic therapy is to mitigate the risk of reinfection. However, restoration of occlusal function can also affect the process of bone healing and remodelling. An in vivo study by Tronstad et al. [42] looked at the relationship between the quality of a root canal filling, a coronal restoration, and the periapical health of teeth that have undergone endodontic treatment. The evaluation criteria utilised to appraise the quality of endodontic treatment and the adjacent structures were similar to those employed in the present study.

Our study had certain limitations. First, assessment of the quality of post and core restorations and apical conditions using two-dimensional images of three-dimensional structures is challenging. Notably, owing to the cross-sectional design of this study, determining the disease activity or healing is not possible, which is a potential limitation. Consequently, these types of studies are considered to have a lower ranking in the hierarchy of evidence levels than longitudinal studies. Furthermore, the use of a cross-sectional design allows for the inclusion of a substantial number of patients, which is challenging to accomplish and regulate in longitudinal investigations. Studies with larger sample sizes may reduce the impact of interpretation bias.

## 5 Conclusions

Within the limitations of this study, it can be concluded that the technical quality of the remaining GP after restoration was comparable to that of previous epidemiological investigations. However, the technical quality of the post was deemed suboptimal. Moreover, insertion of posts in the molars and the use of screwed posts are not recommended and should be not used in contemporary dental procedures. Regrettably, this practice continues to persist within the study population. Upgrading in dental education and training at dental schools and practitioners are warranted to ensure the implementation of recent techniques and materials in clinical practice.

## Ethics approval and consent to participate

The study protocol was approved by the Medical Ethics Committee of the College of Dentistry, University of Ha'il, Saudi Arabia. The requirement for informed consent was waived by the Ethics Committee of the College of Dentistry, University of Hail, Saudi Arabia. All procedures were performed in accordance with the principles of the Declaration of Helsinki.

## Consent for publication

Not Applicable.

## Availability of data and materials

The datasets created and/or analyzed for the current study are not publicly accessible because ethics approval was given on the grounds that only the researchers involved in the study would have access to the identified data; however, they are available from the corresponding author upon reasonable request.

## Funding

Not Applicable.

## CRediT authorship contribution statement

**Moazzy I. Almansour:** Writing – review & editing, Writing – original draft, Visualization, Validation, Supervision, Software, Resources, Project administration, Methodology, Investigation, Funding acquisition, Formal analysis, Data curation, Conceptualization. **Ahmed A. Madfa:** Writing – review & editing, Writing – original draft, Visualization, Validation, Supervision, Software, Resources, Project administration, Methodology, Investigation, Funding acquisition, Formal analysis, Data curation, Conceptualization. **Alanoud N. Alotaibi:** Writing – review & editing, Writing – original draft, Methodology, Investigation, Data curation. **Rawan T. Alturki:** Writing – review & editing, Writing – original draft, Methodology, Investigation, Data curation. **Ahad F. Alshammari:** Writing – review & editing, Writing – original draft, Visualization, Methodology, Investigation.

## Declaration of competing interest

The authors declare the following financial interests/personal relationships which may be considered as potential competing interestsMOAZZY reports was provided by University of Ha'il. None If there are other authors, they declare that they have no known competing financial interests or personal relationships that could have appeared to influence the work reported in this paper.
